# Rates of PCR Positivity of Pleural Drainage Fluid in COVID-19 Patients: Is It Expected?

**DOI:** 10.3390/life14121625

**Published:** 2024-12-08

**Authors:** Hasan Turut, Neslihan Ozcelik, Aysegul Copur Cicek, Kerim Tuluce, Gokcen Sevilgen, Mustafa Sakin, Basar Erdivanli, Aleksandra Klisic, Filiz Mercantepe

**Affiliations:** 1 Department of Thoracic Surgery, Faculty of Medicine, Recep Tayyip Erdogan University, 53100 Rize, Turkey; kerim.tuluce@erdogan.edu.tr (K.T.); gokcen.sevilgen@erdogan.edu.tr (G.S.); 2 Department of Chest Diseases, Faculty of Medicine, Recep Tayyip Erdogan University, 53100 Rize, Turkey; neslihan.ozcelik@erdogan.edu.tr; 3 Department of Medical Microbiology, Faculty of Medicine, Istanbul Medipol University, 34810 Istanbul, Turkey; aysegul.copur@medipol.edu.tr; 4 Department of Anesthesiology, Rize State Hospital, 53020 Rize, Turkey; mustafa.sakin@saglik.gov.tr; 5 Department of Anesthesiology, Faculty of Medicine, Recep Tayyip Erdogan University, 53100 Rize, Turkey; basar.erdivanli@erdogan.edu.tr; 6 Faculty of Medicine, University of Montenegro, 81000 Podgorica, Montenegro; aleksandranklisic@gmail.com; 7 Center for Laboratory Diagnostics, Primary Health Care Center, 81000 Podgorica, Montenegro; 8 Department of Endocrinology and Metabolism, Faculty of Medicine, Recep Tayyip Erdogan University, 53100 Rize, Turkey

**Keywords:** COVID-19, SARS-CoV-2 PCR, pleural fluid, pneumothorax, tube thoracostomy

## Abstract

Background: Tube thoracostomy, utilized through conventional methodologies in the context of pleural disorders such as pleural effusion and pneumothorax, constitutes one of the primary therapeutic interventions. Nonetheless, it is imperative to recognize that invasive procedures, including tube thoracostomy, are classified as aerosol-generating activities during the management of pleural conditions in patients afflicted with COVID-19, thus raising substantial concerns regarding the potential exposure of healthcare personnel to the virus. The objective of this investigation was to assess the SARS-CoV-2 viral load by detecting viral RNA in pleural drainage specimens from patients who underwent tube thoracostomy due to either pleural effusion or pneumothorax. Methods: In this single-center prospective cross-sectional analysis, a real-time reverse transcriptase (RT) polymerase chain reaction (PCR) assay was employed to conduct swab tests for the qualitative identification of nucleic acid from SARS-CoV-2 in pleural fluids acquired during tube thoracostomy between August 2021 and December 2021. Results: All pleural drainage specimens from 21 patients who tested positive for COVID-19 via nasopharyngeal PCR, of which 14 underwent tube thoracostomy due to pneumothorax, 4 due to both pneumothorax and pleural effusion, and 3 due to pleural effusion, were found to be negative for SARS-CoV-2 RNA. Moreover, individuals exhibiting pleural effusion were admitted to the intensive care unit with a notably higher incidence, yet demonstrated significantly more radiological anomalies in patients diagnosed with pneumothorax. Conclusions: The current findings, inclusive of the results from this study, do not furnish scientific evidence to support the notion that SARS-CoV-2 is transmitted via aerosolization during tube thoracostomy, and it remains uncertain whether the virus can be adequately contained within pleural fluids.

## 1. Introduction

COVID-19, originating in Wuhan, China, toward the conclusion of 2019, has had a global impact and was classified as a pandemic by the World Health Organization in February 2020; this disease is attributable to the SARS-CoV-2 virus and is linked to severe respiratory ailments [1]. The epidemic has influenced millions of individuals worldwide in a relatively brief timeframe, a phenomenon exacerbated by the mobility characteristic of contemporary society [2]. The primary mode of transmission for this disease occurs through respiratory droplets and aerosols expelled by infected individuals [3]. Nevertheless, the passage of time has elucidated that the disease manifests in various clinical presentations and systemic repercussions, demonstrating the virus’s capacity to affect not only the respiratory system but also other organs and physiological systems [2,4].

The predominant site of involvement in patients with COVID-19 is the pulmonary parenchyma [3]. Although the incidence of pleural effusion and pneumothorax in these patients is comparatively uncommon, the existing literature provides limited information, primarily consisting of isolated case reports [3,4,5,6,7,8,9]. Based on these scant data, it is estimated that pleural effusion occurs in approximately 5% of COVID-19 patients, while pneumothorax is observed in about 1% of affected individuals [6,7,10,11]. It is posited that the prevalence of these pathologies escalates, particularly in patients experiencing severe clinical manifestations [11]. The etiological factors contributing to pleural effusion include pleural inflammation induced by COVID-19, secondary bacterial infections, or systemic inflammatory responses associated with critical illness; conversely, pneumothorax may infrequently arise spontaneously, though it typically occurs as a consequence of barotrauma in patients undergoing mechanical ventilation [10,11,12]. Given the extensive burden imposed by the COVID-19 pandemic and the proportion of patients requiring mechanical ventilatory support, it is a reasonable expectation that the incidence of both pleural effusion and pneumothorax will experience a global increase throughout the duration of the pandemic.

Tube thoracostomy, a procedure traditionally employed for the treatment of pleural conditions such as pleural effusion and pneumothorax, represents one of the initial therapeutic interventions available [13]. Nonetheless, given that invasive techniques like tube thoracostomy are classified as aerosol-generating during the management of pleural disorders in patients diagnosed with COVID-19, coupled with the detection of SARS-CoV-2 viral RNA in postmortem pleural fluid, the potential exposure risk for healthcare professionals to the virus has emerged as a considerable issue [3,4]. This apprehension is substantiated by prior research indicating that viruses, including hepatitis B virus and HIV, have been identified through aerosolization of biological fluids amid laparoscopic surgical procedures [14,15]. In light of this concern, a range of precautions have been implemented, encompassing the utilization of personal protective equipment to mitigate aerosol-generating procedures during chest tube insertion, as well as the incorporation of filters designed to inhibit viral dissemination [3,4]. Nevertheless, the question of whether pleural fluid harbors SARS-CoV-2 RNA and whether such fluid represents a transmission risk has been addressed only in a limited number of case reports within the existing literature [2,16]. Furthermore, recent reviews have investigated the implications of aerosol generation during surgical interventions in the context of COVID-19 infection; however, no evidence has thus far been established to indicate a correlation between these two variables [11,12]. In contrast, a study examining laparoscopic surgery conducted in Italy revealed the absence of SARS-CoV-2 RNA in 20 swabs collected from various biological fluids [17]. Consequently, while SARS-CoV-2 RNA is typically detected in respiratory tract samples, the viral load and potential for infection within extrapulmonary fluids remain incompletely understood. In this regard, ascertaining the presence of SARS-CoV-2 RNA in the pleural fluid of COVID-19 patients presenting with pleural effusion or pneumothorax and ensuring that this fluid undergoes thorough evaluation for transmission risk is of paramount significance for the safety and health of both patients and healthcare practitioners.

At the time of writing the current study, PUBMED alone cataloged 455,146 publications pertaining to COVID-19. Nonetheless, a limited subset of these, primarily consisting of a few case reports, examined the presence of SARS-CoV-2 RNA in pleural fluids [2,16,17]. As clinicians engaged in the care of patients throughout the pandemic, we, akin to numerous healthcare practitioners, harbored apprehensions regarding the viral load and potential transmission of SARS-CoV-2 in biological fluids such as pleural and peritoneal fluids, extending beyond the conventional droplet and aerosol transmission routes. This concern was particularly pronounced among surgeons and surgical nurses who, despite prevailing challenges, were compelled to persist in performing surgical interventions deemed necessary. Indeed, surgical protocols disseminated during the initial phase of the COVID-19 pandemic advocated for the avoidance of laparoscopic procedures wherever feasible, motivated by apprehensions regarding the chimney effect associated with high-flow intraperitoneal gas leakage during and subsequent to surgical operations, which could heighten the risk of viral dissemination [18]. To address this issue, we conducted SARS-CoV-2 RT-PCR analyses on pleural drainages from COVID-19 patients to ascertain the presence of SARS-CoV-2 RNA in pleural fluid acquired via tube thoracostomy in individuals who developed pleural effusion or pneumothorax, thereby evaluating this fluid with respect to its transmission risk. Understanding the SARS-CoV-2 viral load and associated transmission risk in pleural fluids may yield critical insights for both the safety of healthcare personnel and the clinical protocols governing invasive procedures. Consequently, this investigation aimed to assess the potential for the viral transmission of SARS-CoV-2 during tube thoracostomy by detecting viral RNA in pleural fluid samples.

## 2. Participants and Methods

This investigation represents a prospective, cross-sectional analysis performed on patients diagnosed with COVID-19 at a tertiary university hospital. The cohort comprised patients admitted to the emergency department of our institution between August 2021 and December 2021 who were subsequently identified to have pleural effusion and pneumothorax during their hospitalization in the wards and intensive care unit; only those with a confirmed positive nasopharyngeal COVID-19 PCR test were incorporated into this research. Approval for the study was granted by the Ethics Committee of Recep Tayyip Erdogan University. Participation in this study was entirely voluntary, with all subjects, or in circumstances where the patient was incapacitated due to their medical condition, their guardians who provided signed consent, being included in the analysis. The protocols outlined in the Declaration of Helsinki were rigorously observed throughout the duration of the study. Demographic information of the participants along with biochemical parameters derived from standard pleural fluid analyses were meticulously documented. The sites of intervention (emergency department, wards, and intensive care unit), admission dates, and discharge outcomes following treatment or mortality were systematically assessed. Radiological data, specifically computed tomography (CT) findings, were cataloged according to the CO-RADS classification [19], which included observations of ground glass opacities, consolidations, pleural effusions, and pneumothorax documented in a dataset for each individual patient. The effusion samples obtained from pleural tube drainage fluids and collected from patients via thoracentesis for pleural effusion or tube thoracostomy for pneumothorax underwent COVID-19 PCR testing in the microbiology laboratory.

*Inclusion Criteria*

- Patients exhibiting newly developed pleural effusion following a diagnosis of COVID-19;
- Patients who received treatment for COVID-19 and subsequently required tube thoracostomy due to pleural effusion or pneumothorax.

*Exclusion Criteria*

- Patients possessing a prior history of pleural effusion prior to the current hospital admission who had been evaluated for this condition previously;
- Patients with documented heart failure, cirrhosis, chronic renal failure, and bilateral pleural effusion;
- Individuals younger than 18 years of age;
- Pregnant and lactating patients;
- Patients who refuse to participate in the study or whose legal guardians do not provide consent.

### 2.1. SARS-CoV-2 Real-Time PCR

An indigenous real-time PCR kit was developed in our country based on the protocols declared by international reference centers since the beginning of the pandemic and was used widely across the nation. This study used the real-time “Bio-Speedy^®^ SARS-CoV-2 Double Gene RT-qPCR Kit (Bioeksen Ltd., Istanbul, Türkiye) that was developed with the use of national resources. The analytical and clinical performance of the kit was defined by the Microbiology Reference Laboratories and Biological Products Department of The Ministry of Health, General Directorate of Public Health. The kit has a sensitivity of 99.4% and a specificity of 99%. Viral RNA was obtained using the Biospeedy viral nucleic acid buffer (Bioeksen Ltd., Istanbul, Türkiye), and RT-PCR was performed with the Bio-Speedy^®^ SARS-CoV-2 Double Gene RT-qPCR Kit (Bioeksen Ltd., Istanbul, Türkiye) using primers and probes that target the RNA-dependent RNA polymerase (RdRp) gene fragment on the BioRad CFX 96 System. A standard 20 mL reaction mixture consisting of 5 mL OligoMix, 10 mL 2X Prime ScriptMix, and 5 mL RNA was used. It was processed in a thermal cycler as per the suggestions of the manufacturer for a total of 40 cycles including one cycle each at 52 °C for 3 min and at 95 °C for 10 s, five cycles each at 95 °C for 1 s, and at 55 °C for 12 s, and lastly at 85 °C for 1 s and at 55 °C for 1 s. An OligoMix containing SARS-CoV-2 (N), SARS-CoV-2 (ORF1ab), and internal control (IC) (RNase P) was used. At each run, negative and positive controls were employed to validate the result. Results with CT values ≤ 32 were considered positive.

### 2.2. Statistical Analysis

Data were analyzed using the IBM SPSS Statistics 21.0 (SPSS, Inc., Chicago, IL, USA) software package. Continuous variables are presented as the mean ± standard deviation, while categorical variables were presented as numbers and percentages (minimum and maximum valuable). The comparison of the groups with and without pleural effusion used the student *t*-test for numeric data and the Chi-square test for categorical data. *p* < 0.05 was considered statistically significant.

## 3. Results

In the present investigation, a comprehensive cohort of 21 patients underwent the procedure of tube thoracostomy. Among these individuals, 14 were diagnosed exclusively with pneumothorax, 4 presented with a concomitant occurrence of pneumothorax and pleural effusion, and 3 were identified with isolated pleural effusion. The average age of the participants was recorded as 57 ± 21 years, with 6 (28.5%) identified as female and 15 (71.5%) as male. A distribution analysis revealed that 33% (n = 7) of the patients were assessed in the emergency department, 19% (n = 4) were evaluated in the COVID-19 ward, and 48% (n = 10) were treated in the intensive care unit. Imaging studies via computed tomography (CT) disclosed a ground-glass opacity in 86% of the patients, consolidation in 62%, pleural effusion in 33%, and pneumothorax in 85.7%. The simultaneous presence of both pleural effusion and pneumothorax was documented in four patients (19%). In three instances where pleural fluid was procured through thoracentesis, the characteristics of the pleural fluid were identified as exudative based on Light’s criteria, yielding a pleural fluid/serum LDH ratio of 0.7. The leukocyte composition in pleural effusions associated with COVID-19 exhibited variability; notable distinctions were observed between lymphocytic and neutrophilic predominance. The pleural fluid LDH concentration was found to be significantly elevated in comparison to serum LDH in a singular patient (Table 1).

The diagnosis of empyema was ruled out through positive pleural fluid cultures in one patient who underwent pleural drainage. No microbial proliferation was detected in the culture analysis of pleural fluid specimens from the other two patients. The SARS-CoV-2 PCR assessment yielded negative results in all examined pleural drainage fluids (n = 21). Upon categorizing patients into two distinct groups—those with pleural effusion and those with isolated pneumothorax—the frequency of intensive care unit admissions and the severity of radiological findings were determined to be significantly elevated in the cohort presenting with only pneumothorax. No statistically significant discrepancies were identified between the two groups regarding age, gender, duration of hospitalization, or laboratory metrics (Table 2).

Among the 21 patients incorporated in the study, 11 (52%) succumbed to their condition, while 10 (48%) were discharged with complete recovery. The onset of pleural effusion typically manifested 10–14 days subsequent to the onset of symptoms and within the initial week following hospitalization among COVID-19 patients. This observation implies that pleural effusion tends to occur during the advanced stages of the disease.

## 4. Discussion

In the present investigation, SARS-CoV-2 RNA was meticulously analyzed utilizing RT-PCR methodologies in the pleural fluid of 21 COVID-19 patients who underwent tube thoracostomy due to the presence of pleural effusion or pneumothorax, with no viral RNA being detected in any of the samples. These results suggest that pleural fluid does not pose a risk for the transmission of SARS-CoV-2 and furnish critical data that may alleviate apprehensions regarding the transmission risk during invasive medical procedures involving these patients. In the existing literature, the detection of SARS-CoV-2 RNA in the pleural fluid of COVID-19 patients has been infrequently documented [2,16,17]. For instance, although positivity for viral RNA has been reported in pleural fluid in a limited number of case studies, this occurrence has typically been correlated with a severe progression of the disease and a significantly elevated systemic viral load [12]. Conversely, SARS-CoV-2 RNA was found to be negative in 20 distinct biological fluids (comprising 5 from pleural fluid, 13 from peritoneal fluid, and 2 from bile fluid) examined during laparoscopic surgical interventions at a facility in Italy, analogous to our findings [17]. This phenomenon can be elucidated by the fact that the virus predominantly replicates within alveolar epithelial and bronchial mucosal cells, thereby not permeating the pleural and peritoneal cavities. Anatomical and biological barriers that restrict the ingress of SARS-CoV-2 into the pleural space may serve as significant factors substantiating this observation [17].

According to review articles concerning pleural complications in COVID-19 patients, the incidence of pleural effusion ranges from 5% to 11% [7]. Nonetheless, these review studies accentuate that pleural effusion may arise secondary to comorbidities such as heart failure, renal failure, or complications like pulmonary embolism, rather than as a direct consequence of primary viral infection [7,10]. In our research, additional pathologies or complications such as heart failure, cirrhosis, pulmonary embolism, or hypoalbuminemia were systematically excluded from the assessment of patients presenting with pleural effusion. The inaugural case report of PCR positivity in pleural fluid by Mei et al. [2] and a subsequent investigation by Turrizoni et al. [7] posited that the virus was the primary etiological agent of pleural fluid accumulation, directly provoked by lung parenchymal injury in 3 out of 8 patients. However, the negative PCR results obtained from pleural effusion and pleural drainage samples of individuals diagnosed with pneumothorax contest this hypothesis.

The transmissible nature of the pathology, coupled with the reluctance to undertake invasive interventions, has resulted in the documentation of pleural fluid characteristics in COVID-19 cases in a limited number of studies [9,10,16,20]. Typically, an exudative pleural effusion exhibits a lymphocytic profile attributable to viral pneumonia; nonetheless, a predominance of neutrophils may be observed in the initial stages due to the lymphopenia frequently seen in patients afflicted by COVID-19 [10,11]. Likewise, the infection of viral parapneumonic effusions by secondary bacterial pathogens in later stages also results in a neutrophilic predominance [10]. Among our cohort, three patients presented with exudative pleural effusion, two of whom exhibited lymphocytic predominance, while the third case, which demonstrated growth of Pseudomonas aeruginosa, displayed neutrophilic predominance.

Pleural complications that arise during the follow-up of COVID-19 patients are believed to correlate with prolonged mortality associated with the presence of both pleural effusion and pneumothorax [11,12]. Consistent with the extant literature, the average duration of hospitalization for our patients was 18 days, with a mortality rate recorded at 52% [3,10]. No statistically significant difference in mortality rates was observed between patients with pleural effusion and those with pneumothorax. In instances of pneumothorax identified in COVID-19 patients, pre-existing pulmonary conditions and active smoking were not established as contributing risk factors. In our investigation, the mean age of patients presenting with pneumothorax was 56 years, consistent with the existing literature, and these individuals were devoid of any concomitant comorbidities [12]. A review study established that the interval to diagnosis of pneumothorax varied from 9 to 19.6 days [12]; our findings indicated a mean of 12 days, and in accordance with the literature, a higher incidence of pneumothorax was observed in patients undergoing mechanical ventilation during their follow-up.

In a systematic review conducted by Chong et al., it was determined that the prevalence of the pneumothorax was higher among males, frequently presenting unilaterally and predominantly on the right side [12]. In alignment with this finding, our investigation revealed that 10 out of 14 patients diagnosed with pneumothorax were male, and all instances of pneumothorax were noted to be unilateral; however, in contrast to the aforementioned review, pneumothorax was observed on the right side in 5 patients, while the remainder were located in the left hemithorax. Although the overall incidence of pneumothorax associated with COVID-19 is documented to be as low as 0.3–1% within the existing literature, this figure can escalate to between 12.8–23.8% among critically ill patients necessitating invasive mechanical ventilation (IMV) [11,21]. The occurrence of pleural effusion related to COVID-19 is reported at an approximate rate of 5–10% in the literature [12]. The lower incidence of pleural effusions observed in our cohort can be attributed to various additional factors that may elucidate the presence of pleural effusion in these patients, as well as the fact that the majority of pleural effusion cases were managed conservatively without the need for intervention and thus were excluded from our analysis. Consequently, we required interventional procedures for pleural effusion in a limited subset of patients, which allowed for the inclusion of a restricted number of patients with pleural effusion in our study. The precautions necessitated for invasive procedures in the context of such a high-risk pathological condition necessitate an understanding of the PCR positivity rates of drainage and lavage fluids obtained from the pleural space. A review of the literature reveals various studies aimed at detecting SARS-CoV-2 in diverse clinical specimens; however, we did not encounter any research that performed PCR testing on pleural drainage fluids collected following tube thoracostomy. Only a single case report published by Malik et al. documented a positive result in the RT-PCR assay of a pleural fluid sample acquired via thoracentesis from a patient exhibiting viral parapneumonic pleural effusion. Notably, the nasopharyngeal swab sample from this patient also yielded a positive result in the RT-PCR COVID-19 test [8]. In a manner analogous to the findings presented in our study, Fabbri et al. reported the absence of SARS-CoV-2 RNA in pleural fluid samples from five patients [17].

A study carried out by Wang et al. involving various samples analyzed 1070 specimens from 205 COVID-19 patients; the bronchoalveolar lavage fluid samples exhibited the highest positivity rate (14 of 15, 93%), followed by sputum (72 of 104; 72%), nasal swab (5 of 8; 63%), fibrobronchoscope brush biopsy (6 of 13; 46%), pharyngeal swab (126 of 398; 32%), stool (44 of 153; 29%), and blood (3 of 307; 1%). Notably, none of the 72 urine samples tested positive in this study [22]. As previously mentioned, in a study conducted in Italy involving COVID-19 patients who underwent laparoscopic surgical procedures, none of the five pleural fluid, 13 peritoneal fluid, and 2 bile fluid swab samples were found to be positive for SARS-CoV-2 RT-PCR [17]. This observation implies that distinct biological fluids may exhibit varying viral loads, and it is even possible that certain anatomical areas have not been exposed to the virus.

Pleural complications associated with viral infections remain inadequately addressed in the existing literature, particularly in the context of extensive case series utilizing satisfactory scales [23]. Furthermore, the available data is insufficient to facilitate a robust conclusion regarding the viral load present within the pleural space during COVID-19 infections. Consequently, it is posited that our investigation may illuminate this matter and contribute to the formulation of innovative ideas and methodologies. It should be acknowledged that the authors have not overlooked the possibility that the lack of PCR positivity among all participants in our study could be attributable to the PCR technique employed. Conversely, the outcomes of this research also mitigate apprehensions regarding the necessity to refrain from tube thoracostomy in cases of pleural effusion or pneumothorax. Nevertheless, standard infection control protocols must be diligently upheld to safeguard healthcare practitioners. Despite the negative RT-PCR findings, the potential for pleural fluid to harbor alternative infectious agents during aerosolization should not be disregarded. Additionally, certain strengths and limitations inherent to our study warrant consideration. Notably, the strengths of our study encompass its status as one of the few prospective investigations that systematically examines viral load within pleural fluid in patients diagnosed with COVID-19. However, the study is not devoid of limitations. The most significant limitation pertains to the modest sample size. Furthermore, there exists a multitude of factors that could account for negative RT-PCR results in pleural fluid, which may originate from both biological and technical sources. Primarily, SARS-CoV-2 exhibits preferential replication within the respiratory tract, specifically targeting alveolar epithelial cells [24]. Hence, the anatomical barrier separating the pleural space from the alveolar compartments may have restricted the virus from infiltrating the pleural fluid [10]. Additionally, pleural effusion associated with COVID-19 may arise from inflammatory or immunological responses in the absence of active viral replication within the pleural fluid [12]. A further consideration is that systemic viral load may diminish during the later phases of the disease, thereby evading detection in extrapulmonary regions. Moreover, inflammatory processes occurring within the pleural space may inhibit viral replication by instigating the degradation of viral particles. Technical factors include the possibility that the viral load in the pleural fluid may have fallen below the detection threshold of RT-PCR when juxtaposed with respiratory tract samples; circumstances such as inadequate pleural fluid volume or unsuitable transport conditions; suboptimal reaction environments or the presence of inhibitors; and critically, given that pleural fluids may exhibit differing biochemical characteristics compared to respiratory tract samples, the test may not yield optimal performance in these fluids. The quantification of nucleic acids via reverse transcriptase-polymerase chain reaction (RT-PCR) constitutes the most sensitive diagnostic modality for coronavirus disease 2019 [25]. Nonetheless, various publications in the literature have reported instances of false-negative results [26]. To address these challenges, forthcoming studies should be undertaken to develop more sensitive testing methodologies or supplementary validation studies and protocols aimed at detecting SARS-CoV-2 in biological fluids, considering all aforementioned factors. Furthermore, it is imperative that future investigations examine potential immunological and inflammatory markers, even in instances where SARS-CoV-2 is not identifiable in pleural fluid. Conversely, regarding alternative interpretations of our findings, we underscore the significance of this study as foundational comparative data to ascertain whether novel strains of the COVID-19 pathogen, which have evolved due to mutations over time, can induce pleural effusion and the associated morbidity in affected individuals, as well as to forecast clinical risk factors and radiological indicators pertinent to pleural effusion accumulation in these patients. In summary, the detection of SARS-CoV-2 RNA’s absence in pleural fluid among COVID-19 patients yields a promising implication for the safety of invasive interventions and clinical methodologies. These results may enhance the formulation of protective strategies for healthcare practitioners, particularly surgeons and surgical nurses operating within intensive care settings who are required to conduct invasive procedures.

## 5. Conclusions

There exists a lack of conclusive scientific evidence within the current literature indicating that SARS-CoV-2 is detectable in specimens obtained through tube thoracostomy due to pleural effusion or pneumothorax, nor are there sufficient data to assert that such presence elevates the risk of transmission to healthcare personnel via aerosolization. Nevertheless, it is imperative to acknowledge that the methodologies employed to evaluate viral load in biological fluids (such as assay types, sample dimensions, and the immunological status of the patients) may constitute confounding variables that could potentially skew the findings. The correlation between elevated viral loads in symptomatic individuals and augmented viral load within the pleural cavity remains ambiguous and necessitates further empirical exploration. The implementation of cautious strategies during the pandemic has resulted in the cessation of minimally invasive interventions in favor of alternative approaches. Nonetheless, the postponement of critical procedures like tube thoracostomy may heighten the likelihood of adverse complications for patients and detrimentally impact clinical outcomes. The utilization of appropriate personal protective equipment is paramount to safeguarding the welfare of healthcare practitioners. Consequently, additional research is essential to formulate more comprehensive guidelines and procedural frameworks that will achieve an equilibrium, ensuring both patient safety and the safeguarding of healthcare professionals.

## Figures and Tables

**Table 1:** Biochemical analysis of pleural effusions and SARS-CoV-2 evaluation from pleural fluid.

Pleural Fluid Characteristics			
Parameter	Patient 1	Patient 2	Patient 3
Appearance	Clear	Clear	Blurred
Color	Yellow	Yellow	Yellow–Green
Total protein, g/dL	2.3 g/dL	3 g/dL	4.7 g/dL
Lactate dehydrogenase, U/L	315 U/L	189 U/L	6770 U/L
WBC count	120/mcl (mononucleated cells)	480/mcl (polymorphonucleated cells)	3200/mcl (polymorphonucleated cells)
Microbiology	Negative	Negative	Pseudomonas aeruginosa
Glucose, mg/dL	115 mg/dL	133 mg/dL	10 mg/dL
SARS-CoV-2 (RT-PCR)	Negative	Negative	Negative

**Table 2:** Comparison of the patients with/without pleural effusion.

	Pleural Effusion (n: 7)	Pneumothorax (Only px) (n: 14)	*p*
Age (year)	61 ± 27	56 ± 18	0.649
Gender (F/M)	3/4	4/10	0.357
Hospitalization (day)	17.7 ± 12.2	20.0 ± 8.8	0.634
Follow-up (ward/intensive care unit)	7/0	5/9	0.003 *
Survival (discharge/exitus)	4/3	5/8	0.423
CO-RADS	2.3 ± 1.3	3.8 ± 1.0	0.010 *
WBC (/µL)	8343 ± 5233	14,016 ± 7875	0.129
Neutrophile (/µL)	8995 ± 7317	12,294 ± 7340	0.350
Lymphocyte (/µL)	1115 ± 712	1137 ± 1164	0.964
Eosinophile (/µL)	22 ± 22	46 ± 87	0.486
Trombocyte (/mlx1000)	262 ± 154	253 ± 167	0.611
Creatinine (mg/dL)	0.85 ± 0.28	1.22 ± 0.98	0.346
LDH (U/L)	594 ± 102	851 ± 120	0.067
CRP (mg/L)	134 ± 102	59 ± 64	0.061
D-dimer (µg FEU/mL)	10.3 ± 17.2	2.5 ± 2.2	0.566
Ferritin (ng/mL)	1602 ± 2625	841 ± 422	0.328
Procalcitonin	0.44 ± 0.21	2.07 ± 2.91	0.639

* *p* < 0.05 is significant.

## Data Availability

All data generated or analyzed during this study are included in this article. The data will be available upon reasonable request (contact persons: filiz.mercantepe@saglik.gov.tr or hasan.turut@erdogan.edu.tr).
